# Exploring molecular variation in *Schistosoma japonicum* in China

**DOI:** 10.1038/srep17345

**Published:** 2015-12-01

**Authors:** Neil D. Young, Kok-Gan Chan, Pasi K. Korhonen, Teik Min Chong, Robson Ee, Namitha Mohandas, Anson V. Koehler, Yan-Lue Lim, Andreas Hofmann, Aaron R. Jex, Baozhen Qian, Neil B. Chilton, Geoffrey N. Gobert, Donald P. McManus, Patrick Tan, Bonnie L. Webster, David Rollinson, Robin B. Gasser

**Affiliations:** 1 The University of Melbourne, Pathogen Genomics and Genetics Program, Parkville, Victoria 3010, Australia; 2 ISB (Genetics and Molecular Biology), Faculty of Science, The University of Malaya, Kuala Lumpur 50603, Malaysia; 3 Structural Chemistry Program, Eskitis Institute for Drug Discovery, Griffith University, Brisbane, Queensland 4111, Australia; 4 Department of Biology, University of Saskatchewan, Saskatoon S7N 5E2, Canada; 5 QIMR Berghofer Medical Research Institute, Brisbane, Queensland 4006, Australia; 6 Genome Institute of Singapore, 60 Biopolis Street, Singapore 138672, Republic of Singapore; 7 Cancer and Stem Cell Biology, Duke-NUS Graduate Medical School, Singapore 138672, Republic of Singapore; 8 The Natural History Museum, London SW7 5BD, United Kingdom

## Abstract

Schistosomiasis is a neglected tropical disease that affects more than 200 million people worldwide. The main disease-causing agents, *Schistosoma japonicum*, *S. mansoni* and *S. haematobium*, are blood flukes that have complex life cycles involving a snail intermediate host. In Asia, *S. japonicum* causes hepatointestinal disease (schistosomiasis japonica) and is challenging to control due to a broad distribution of its snail hosts and range of animal reservoir hosts. In China, extensive efforts have been underway to control this parasite, but genetic variability in *S. japonicum* populations could represent an obstacle to eliminating schistosomiasis japonica. Although a draft genome sequence is available for *S. japonicum*, there has been no previous study of molecular variation in this parasite on a genome-wide scale. In this study, we conducted the first deep genomic exploration of seven *S. japonicum* populations from mainland China, constructed phylogenies using mitochondrial and nuclear genomic data sets, and established considerable variation between some of the populations in genes inferred to be linked to key cellular processes and/or pathogen-host interactions. Based on the findings from this study, we propose that verifying intraspecific conservation in vaccine or drug target candidates is an important first step toward developing effective vaccines and chemotherapies against schistosomiasis.

Schistosomiasis is a neglected tropical disease that still affects more than 200 million people in 70 countries, resulting in a burden of at least 3.31 million disability-adjusted life years^1^,^2^. The main disease-causing agents are the blood flukes *Schistosoma japonicum*, *S. mansoni* and *S. haematobium*, which all have complex life cycles involving a snail intermediate host^3^. Schistosomiasis japonica has affected human populations in many parts of Asia, including the People’s Republic of China, Indonesia and the Philippines^1^,^2^. In China, it has been one of the major hepatointestinal diseases in this region for more than 2,100 years^4^, and is particularly challenging to control due to the wide distribution of its snail hosts (genus *Oncomelania*) and the range of domestic and wild mammals that act as reservoirs for human infection^5^,^6^.

Since the implementation of the National Schistosomiasis Control Program in the mid 1950s, the number of reported cases of this disease in China has decreased significantly^5^,^7^. This reduction has been due to changes in China’s health policy, leading to the implementation of snail control (1950s-early 1980s), mass drug administration programmes (mid 1980s to 2003) and subsequent, integrated control regimens (2004 onward) to break the transmission cycle^5^,^8^,^9^. No vaccine is available for use in humans, and the reliance on praziquantel alone to treat/control schistosomiasis over a long period of time carries a risk of the emergence of drug resistance^10^,^11^. In spite of the availability of extensive genomic resources for schistosomes^12^,^13^,^14^, little is known about genome-wide changes that take place over time and space in *S. japonicum* populations, or molecular variation within *S. japonicum* from humans and different animal hosts. Clearly, more genomic information is required for *S. japonicum* populations from distinct geographical regions, particularly those that display distinct biological and ecological characteristics^15^,^16^. Furthermore, genetic diversity and changes in schistosome populations which are under pressure from repeated treatment with drugs, such as praziquantel, and also environmental biophysical effects (i.e. anthropogenic and environmental change) could represent an obstacle to the sustained control or elimination of schistosomiasis^17^,^18^,^19^,^20^. Advanced tools are needed to detect and quantify genetic differences and changes in schistosome populations, and to monitor the spread of genetic variants that might affect control strategies. With extensive and prolonged use of only one main drug to combat schistosomiasis, resistance against praziquantel is a distinct possibility^11^, and new genetic variants could represent a crucial point of vulnerability for any future intervention strategy.

Previous population studies of *S. japonicum* have been limited to relatively small numbers of genetic loci, largely due to a lack of comprehensive genomic sequence data sets for this blood fluke. For instance, microsatellite, mitochondrial (mt) and enzymatic markers were used to reveal genetic variation among isolates of *S. japonicum* from various regions in China and surrounding, coastal islands^21^,^22^,^23^,^24^,^25^,^26^, or to identify genetic bottlenecks in laboratory strains of this parasite^27^. Although these observational studies have been informative, none of them tightly linked genotype to biological traits of the parasite, such as infectivity, pathogenicity and/or immunogenicity.

The advent of high throughput sequencing technologies^28^,^29^ and the availability of a draft genome for *S. japonicum*^13^ have paved the way toward genome-wide studies of natural and laboratory-adapted populations of *S. japonicum* from humans and reservoir hosts. To this end, we undertook here the first deep genomic exploration of various *S. japonicum* populations from China to reveal their systematic relationships as well as considerable genetic variation between some populations, with an impact on numerous genes associated with key metabolic and signalling pathways, cellular processes and/or pathogen-host interactions.

## Results

**Figure 1:**
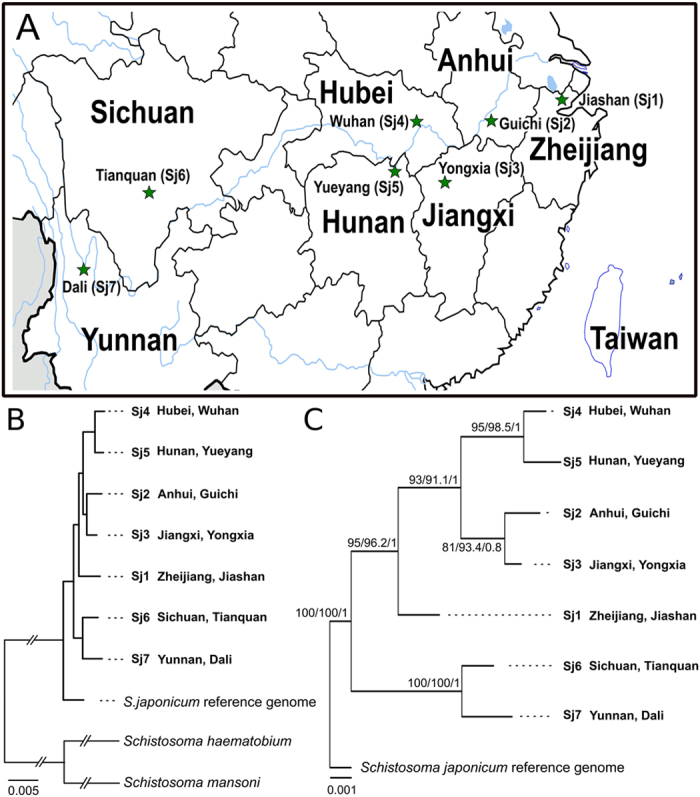
Phylogenetic relationships of seven *Schistosoma japonicum* populations from different parts of China. (**A**) Map indicating the provenance of populations, and their relationships based on Bayesian inference (BI) analysis of (**B**) nucleotide sequence data representing 4,333 protein-encoding single copy orthologs (SCOs) or (**C**) four exonic regions within SCOs (designated Sjp_0006080, Sjp_0009700, Sjp_0068320 and Sjp_0102280). The topology of these BI trees (**B,C**) are the same as those obtained for independent analyses using the maximum parsimony (MP) and maximum likelihood (ML) methods. Absolute nodal support was achieved using each tree building method (**B**). Nodal bootstrap or posterior probability values are indicated in the following order: ML/MP/BI (**C**). Map was modified from https://commons.m.wikimedia.org/wiki/File:China_Heilongjiang_Shuangyashan.svg, and was originally created by Joowwww under the creative commons licence [ http://creativecommons.org/licenses/by-sa/3.0/legalcode] and distributed via Wikimedia Commons.

We used an Illumina-based sequencing approach to produce mitochondrial (mt) and nuclear genomic data sets from genomic DNA samples from *S. japonicum* (Supplementary Fig. 1). This effort yielded 15.5 to 19.8 Gb of high quality genomic sequence data (NCBI BioProject accession no. PRJNA286685) for each of the seven study populations, corresponding to 39- to 50-fold coverage of a reference nuclear genome for *S. japonicum* (Supplementary Table 1). These data were utilised to estimate genetic diversity among *S. japonicum* populations from seven provinces in China (Fig. 1A).

### Assessing *S. japonicum* phylogeny using mitochondrial genomes

First, we *de novo*-assembled the mt genomes of individual *S. japonicum* populations (Supplementary Table 2; NCBI GenBank accession nos. KR855668-KR855674) from 1.3–3.3 million paired-end reads, annotated each genome and compared each set of 12 mt protein-encoding genes to those of published mt genomes (Supplementary Table 2) to assess the phylogenetic informativeness of aligned, concatenated nucleotide and amino acid sequence data sets. At the nucleotide level, 7,841 of 10,341 alignment positions were invariable, and 148 (1.43%) were phylogenetically informative (Table 1). At the amino acid level, 2,375 of 3,438 positions were invariable, but only 39 (1.13%) were informative (Table 1). Phylogenetic trees constructed using Bayesian inference (BI; nucleotide and amino acid) and maximum parsimony (MP; nucleotide only) methods revealed two well-supported clades (Supplementary Fig. 2): one including populations Sj6 (Tianquan, Sichuan) and Sj7 (Dali, Yunnan) from provinces in Western China, and the second with Sj1 (Jiashan, Zhejiang), Sj4 (Wuhan, Hubei) and Sj5 (Yueyang, Hunan) from provinces in Eastern China. These results, however, were inconsistent with those obtained by maximum likelihood (ML; nucleotide and protein) and maximum parsimony (MP; protein) analyses, particularly using the aligned mt protein sequence data set. The clustering of other populations, including Sj2 (Guichi, Anhui) and Sj3 (Yongxia, Jiangxi) from Central China, were not well supported (nodal support: <0.8 or <80%) in analyses using any of the three tree-building methods, precluding further interpretation (Supplementary Fig. 2). This lack of resolution led us to explore genetic variation in nuclear genomic data sets among the seven *S. japonicum* populations.

### Assessing nuclear genomic variation

**Figure 2:**
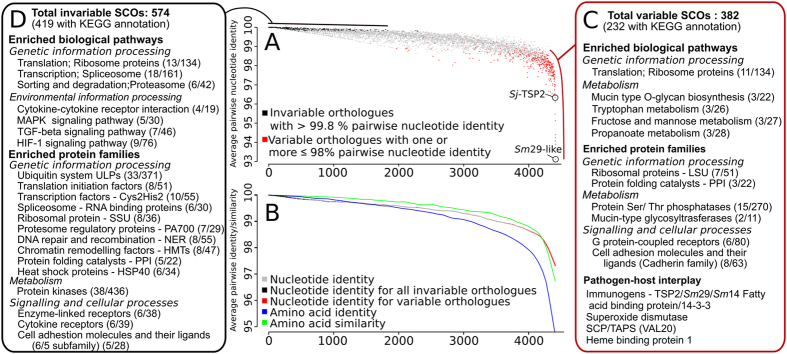
Sequence conservation and variation in single copy orthologs (SCOs) among seven distinct populations of *Schistosoma japonicum*. (**A**) Ranked SCOs, according to pairwise nucleotide sequence identities across coding domains. (**B**) Locally weighted linear regression (LOWESS) analysis of pairwise nucleotide identity, and amino acid identity and similarity among SCOs, ranked according to pairwise nucleotide sequence identities. Invariable SCOs, with >99.8% pairwise nucleotide identity among all populations, are indicated/boxed in black (left). Variable SCOs with one or more pairwise nucleotide identities ≤98% are indicated/boxed in red (right). Significantly enriched Kyoto Encyclopedia of Genes and Genomes (KEGG) biological pathways and protein families as well as proteins involved in the pathogen-host interplay and other biological processes among (**C**) invariable and (**D**) variable SCOs are listed.

We mapped the sequence reads derived from individual populations to a reference nuclear genomic sequence of *S. japonicum* (designated here as SjRef; Bioproject accession no. PRJEA34885)^13^. Overall, 82.6 to 88.0% of all reads mapped to this draft reference genome, with ~95% of these mapping as pairs (Supplementary Table 4). Excluding ambiguous positions (i.e. Ns in SjRef), 6,879,937 to 7,509,073 single nucleotide polymorphisms (SNPs) were recorded in individual populations (Table 2), of which 69% (n = 4,767,718 to 5,196,811), 26% (n = 1,784,457 to 1,948,205) and 1.6% (n = 107,446 to 121,775) were within intergenic, intronic and protein-encoding regions, respectively. For individual *S. japonicum* populations, 55,316 to 64,473 non-synonymous and 51,389 to 57,725 synonymous SNPs were identified in coding domains (Table 2). Employing published genomes, we identified 4,413 single-copy orthologs (SCOs) that were common to *S. japonicum*^13^, *S. haematobium*^14^ and *S. mansoni*^30^. Of the 4,413 single-copy orthologs (SCOs), 697,639 to 768,044 intronic, and 37,273 to 42,333 exonic SNPs were identified in protein-encoding gene regions, with 17,035 to 19,931 non-synonymous and 19,723 to 22,402 synonymous SNPs in individual *S. japonicum* populations (Table 3). For all SCOs, on average, 6.4 SNPs were detected in *S. japonicum* per kb of coding sequence (Table 3). The effect of nucleotide polymorphisms in SCOs on variation in the inferred proteins varied considerably; and, variation was reduced by an accumulation of synonymous mutations (recorded as amino acid identity) or mutations substituting amino acid residues with conserved chemical properties (recorded as amino acid similarity) (Fig. 2B and Supplementary Table 5).

Of all 4,413 SCOs, 382 exhibited >2% nucleotide variation between or among populations (Fig. 2). These variable SCOs, 70.2% of which were functionally annotated (Supplementary Table 5), were significantly enriched for functions relating to genetic information processing (ribosomal translation) or metabolic pathways (i.e. glycan biosynthesis and metabolism; amino acid and carbohydrate metabolism) (Fig. 2C; Supplementary Table 6). These SCOs were also significantly enriched for genetic information processing, such as the large subunit of ribosomal protein (see Supplementary Table 7) and the peptidyl prolyl isomerase (PPI) protein folding catalysts, metabolism (protein phosphatases and glycosyltransferases) and cellular signal processing (G protein-coupled receptors [GPCRs] as well as representatives of the cadherin cell adhesion molecule family) (Fig. 2C; Supplementary Tables 6 and 7). By contrast, 574 SCOs each shared >99.8% nucleotide sequence identity between or among all *S. japonicum* populations, and were thus designated invariable (Fig. 2D). These invariable SCOs, 87.8% of which were functionally annotated (Supplementary Table 5), were significantly enriched for genes associated with genetic information processing (ubiquitin-proteasome complex, ribosomal translation and spliceosome) and environmental information processing [transforming growth factor-β (TGF-β), mitogen-activated protein kinase (MAPK) and hypoxia-inducible factor-1 (HIF-1) signalling and cytokine-cytokine receptor interaction] pathways (Fig. 2D). These invariable SCOs were also significantly enriched for protein families associated predominantly with genetic information processing (ubiquitin-proteosome systems, transcription and translation factors, spliceosome, small subunit ribosomal proteins, DNA repair and remodelling proteins, PPI protein folding catalysts and heat shock proteins), metabolism (protein kinases) or cellular signal processing (cytokine receptors and cell adhesion molecules) (Fig. 2D; Supplementary Table 6).

### Genetic variability linked to host response and disease intervention

**Figure 3:**
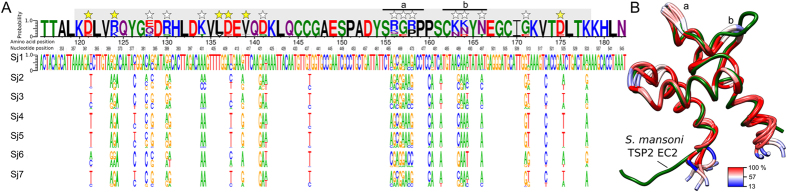
Sequence variability in the extracellular 2 domain (EC2) of the *Schistosoma japonicum* tetraspanin 2 ortholog (*Sj*-TSP2) among seven distinct populations. (**A**) Nucleotide logos represent the frequency of base calls for each population in sites containing single nucleotide polymorphisms (SNPs). Amino acid logos representing the consensus sequence for all seven populations. SNPs leading to a similar (yellow star) or distinct (white star) change of the translated amino acid are indicated. Each amino acid logo is coloured according to its chemical characteristics; polar residues (G, S, T, Y & C) are green, neutral (Q & N) are purple, basic (K, R & H) are blue, acidic (D & E) are red and hydrophobic (A, V, L, I, P, W, F & M) are black. The extracellular 2 (EC2) domain is highlighted in grey. (**B**) Comparison of consensus *Sj*-TSP2-EC2 structures, modelled using the resolved protein structure of *Sm*-TSP2-EC2 (labelled green; RCSB accession number: 2M7Z) and highlighting structural changes (a & b) in the head region associated with the consensus amino acid sequence composition of each *S. japonicum* isolate. Proteins structures (*S. japonicum*) are coloured by percentage amino acid conservation among consensus protein translations.

Pairwise comparisons revealed nucleotide sequence variation of >2% in SCOs encoding structural proteins, molecules recognised to play important roles in regulating or modulating definitive host responses, and known immunogens (Fig. 2C and Supplementary Table 5). Variable structural proteins of cells included five cadherin/protocadherin-like molecules, dynein and actophorin and annexins (Supplementary Table 5). Variable proteins inferred to be involved in the pathogen-host interplay included a disulphide isomerase^31^, a thioredoxin^32^, a venom allergen-like (VAL20) protein^33^, heme-binding protein 1^34^ and an extracellular superoxide dismutase^35^. Known immunogens included *Sm*14-like (fatty acid binding protein), *Sm*29-like^36^ and four tetraspanins (Fig. 2 and Supplementary Table 8). Sequence variability among some members of the tetraspanin protein family of *S. japonicum* was similar to a previous observation^37^ and was detected principally within *Sj*25 and the surface-exposed extracellular domain 2 (EC2) of the TSP2 ortholog (i.e. *Sj*-TSP2-EC2; Fig. 3, Supplementary Fig. 3 and Supplementary Table 8)^37^. *Sj*-TSP2-EC2 is encoded by a single SCO, and displays considerable nucleotide sequence variation (93.8–98.4%, respectively) within *S. japonicum* (Fig. 3A and Supplementary Table 8). A comparison of *Sj*-TSP2-EC2 domains, modelled using the resolved tertiary structure template of *Sm*-TSP2-EC2^38^ (coverage: 96%; root-mean-square deviations between backbone atomic positions: ~2.4 Å), revealed “stem” regions that mediate contact with the plasma membrane^38^ and are structurally conserved between *S. mansoni* and all seven *S. japonicum* isolates (Fig. 3B). The “head region” of *Sj*-TSP2 is stabilised by two strictly conserved disulphide bridges^38^. However, mostly the surface-exposed amino acid residues in the head region are variable (Fig. 3Bb) in this TSP2 moiety between *S. japonicum* populations and between schistosome species. Compared with *Sm*-TSP2, the *Sj*-TSP2 EC2 domain lacks four residues, leading to a loss of the exposed hydrophobic patch in the head region^38^ (Fig. 3Bb). Other notable differences between *S. japonicum* populations include variation in features that likely affect protein-protein interactions, such as the change of surface electrostatics (K28T, D35S and K57N) and alterations that increase flexibility and thus allow for structural changes (P52R). In addition to variation in *Sj*-TSP2 was nucleotide sequence variability in tetraspanin-enriched-microdomain (TEM)^38^-associated proteins, including calpain (98.0–98.9%), annexin (97.4–100%) and an *Sm*29-like molecule (91.8–93.6%) (Supplementary Table 8). Importantly, variation in the *Sm*29-like protein was observed downstream of the N-terminal signal peptide and upstream of the C-terminal hydrophobic transmembrane domain (Supplementary Fig. 3), which has been used to assess immunoprotection in animals against *S. mansoni* infection^39^. Interestingly, there was a positive correlation (0.794) in sequence similarity between the *Sj*-TSP2 and the *Sm*29-like proteins within individual *S. japonicum* populations, suggesting that the evolution of these TEM-associated proteins might be linked.

### Robust phylogenomic reconstruction using nuclear data sets

We explored the phylogenomic relationships of the seven *S. japonicum* study populations, to address current limitations of using small mt or microsatellite DNAs. To do this, we used sequence data sets representing thousands of SCOs, employing the genomes for *S. japonicum*^13^, *S. haematobium*^14^ and *S. mansoni*^30^ as references (Table 1 and Fig. 1B). At the nucleotide level, an alignment of 4,333 of all 4,413 SCO sequences identified 9,947,586 homologous characters, 8,149,863 of which were invariant, and 925,719 (9.3%) of which were variable and phylogenetically informative (Table 2). At the amino acid level, 2,613,069 of 3,315,862 positions were invariant, and 335,882 (10.1%) informative (Table 1). These numbers of informative characters were ~10-fold greater than for mt data sets (cf. Table 1). The trees built from the nuclear data sets (representing 4,333 SCOs) using BI, ML and MP methods produced consensus trees with consistent topology and strongly supported (1.0 or 100%) clades, and unequivocally resolved the relationships among populations from Western (Sj6 and Sj7), Central (Sj4 and Sj5) and Eastern (Sj2 and Sj3) China. The population Sj1 was the farthest east and was basal to those from Eastern China (Fig. 1B). Finally, we sought to define a subset of SCOs containing variable coding regions with conserved flanking sequences within exons, in which primers could be designed and used in future PCR-coupled mutation scanning^40^ and/or sequencing analyses for large-scale population investigations. In total, we identified 662 SCO regions in which such primers could be designed across all seven *S. japonicum* populations. By conducting an exhaustive search, four of these SCO regions (Supplementary Table 9) had an adequate signal to reproduce (using BI, ML and MP; see Fig. 1C) trees, whose topology and nodal support values were consistent with those of the final consensus tree constructed using the complete SCO set (cf. Fig. 1B).

## Discussion

Although previous mitochondrial and microsatellite DNA studies^22^,^23^,^27^,^41^,^42^ had provided some insight into population variation in *S. japonicum*, nucleotide variation was limited^24^,^25^,^26^,^43^, often resulting in relationships with limited statistical support. In this study, our initial aim was to assess the utility of large mitochondrial and nuclear genomic sequence data sets to explore molecular variation within and among populations of *S. japonicum*. The ability to explore such variation in schistosome populations on a genome-wide scale provides a unique opportunity to link genetic variation to genes or gene products associated with important biological and/or disease traits, which has important implications for understanding schistosomiasis, its epidemiology and possibly for its control.

Through the sequencing and analyses of seven new and all publicly available mt genome sequences of *S. japonicum*^44^, we confirmed that mitochondrial data sets lacked sufficient signal to reliably establish relationships among populations, consistent with previous findings^24^,^25^,^43^,^44^. In contrast, we showed that a vast array of single-copy protein-encoding genes (SCOs) in the genome provides a rich source of neutral and adaptive genetic markers^45^.

The phylogenetic analyses of nuclear SCO data suggest that *S. japonicum* initially colonised and “stabilised” in the western valleys of the Sichuan and Yunnan provinces, in accord with their snail intermediate host(s)^43^. They also indicate that *S. japonicum* radiated eastwards, and established as distinct groups in central and eastern provinces, along the Yangtze River. Taken together, we contend that the biogeographic framework constructed here provides a first, robust foundation for large-scale investigations of molecular variation in *S. japonicum* in China and/or other parts of Asia, such as the Philippines^26^,^41^ and Japan^26^, and a basis for future population genetic or biogeographic studies. For laboratories without the budget and facilities to undertake genomic sequencing, the four informative SCO loci identified here (and able to be used to reproduce the consensus tree; Fig. 1B) are expected to be useful for systematic and/or population genetic studies. However, these markers need to be sequenced from large numbers of individual female and male worms representing different populations of *S. japonicum*, in order to assess whether they are neutral or adaptive, and to establish their suitability for particular applications^45^.

We elected to investigate single copy orthologs (SCOs) shared by at least three schistosome species, to be able draw comparisons among protein homologs of known biological relevance (Fig. 2). While the majority of SCOs could be annotated, interestingly, almost 30% of proteins encoded by variable SCOs lacked a functional annotation. This finding is not surprising and consistent with previous studies^12^,^13^,^14^,^46^,^47^,^48^, demonstrating that flatworms differ substantially genetically, and are evolutionarily very distant, from organisms whose genomes are almost fully characterised, and whose gene sets are functionally annotated. In spite of technical challenges, there is major merit in finding a reliable method(s) to annotate presently uncharacterised genes of *S. japonicum* and other schistosomes, as they are believed to play important organism- or species-specific roles.

By focusing on the use of coding regions, we aimed to assess variation in any exon of any SCO of *S. japonicum* with a recognised link to a phenotypic trait, such as host affiliation^16^,^41^, infectivity^49^, pathogenicity, praziquantel susceptibility or resistance^50^, antigenicity or immunogenicity^51^,^52^, and/or to predict how a particular selection pressure might impact on the genotype and/or phenotype of a worm. The power of such an approach is not only in its ability to explore molecular variation within and among worm populations, but, more importantly, among individuals (irrespective of developmental stage) within and among worm populations.

Interestingly, we showed that sequence polymorphism in selected proteins (*Sj*-TSP2 and *Sm*29-like, which are predicted to be essential for tegument integrity and are highly antigenic^39^,^53^) varies considerably among populations, suggesting that it might affect protein structure and thus influence levels of immunogenicity^36^,^54^,^55^,^56^. Based on information for *S. mansoni*^38^,^57^, *Sj*-TSP-2 likely mediates dynamic processes occurring at the tegumental surface and maintains tegumental integrity, and is also a promising vaccine candidate^53^. The finding that *Sj*-TSP2-EC2 (encoded by an SCO) varies considerably in *S. japonicum* agrees with a previous study^55^ indicating that allelic variation results in protein isoforms, but contrasts the hypothesis that *S. japonicum* encodes multiple *tsp2* genes^54^. The variation detected here is suggested to relate to an ability of *S. japonicum* to infect a substantially broader host range than either *S. mansoni* or *S. haematobium*^55^,^58^. Although it is not known how TSP2 interacts with host molecules or immune system, it is understood that calpain, actin, annexin and *Sm*29 all associate closely with *Sm*-TSP2 in tetraspanin-enriched-microdomains (TEMs)^38^. In *S. mansoni*, this association has been proposed to underpin the success of *Sm*-TSP2, annexin and *Sm*29 as vaccine candidate molecules^36^,^39^,^53^,^59^, and might be explained by an induction of an immunogenic host response that disrupts the integrity of TEMs, leading to the destruction of the tegument and subsequent death of the parasite^38^. Interestingly, here, we detected sequence variation in TEM-associated proteins of *S. japonicum*, including a positive correlation between genetic variation in *Sm*29-like and *Sj*-TSP2 molecules. We propose that sequence variation in these molecules might explain inconsistent results (i.e. variable levels of protection, if any) in some vaccination experiments, particularly if the vaccine molecules (i.e. variants of *Sj*-TSP2-EC2) differ in sequence (particularly in the protective epitope/s) from the homolog present in the parasite used for the challenge infection^54^,^55^,^60^. To date, a limited survey of six *S. mansoni* individuals from Kenya suggested that sequence polymorphism within the *Sm*-TSP2-EC2 domain might be less than for *Sj*-TSP2-EC2^61^; however, future studies using large-scale genomic data sets of individuals across a broader geographic range are needed to test this proposal.

Therefore, based on the present findings, we support recommendations^62^,^63^ that, in addition to assessing variation between species^64^,^65^, comprehensive assessments of intraspecific conservation in immunogens or their protective epitopes should precede the research and development of any schistosome vaccine, in order to provide an informed position and some confidence that a vaccine would be efficacious in the field. Although our focus here was principally on immunogenic molecules, similar considerations would apply to targets for the development of new anti-schistosomal drugs. Importantly, the genome-wide approach established here should be applicable to a wide range of eukaryotic pathogens for the analysis of genetic variability. Clearly, neutral SCO markers would have advantages for estimating haplotype diversity and population size, and could provide unbiased estimates of random processes, such as genetic drift. On the other hand, adaptive (non-neutral) markers should have practical applications, for example, to the identification of disease-causing genes or other genes that link phenotype to genotype across different environmental conditions.

## Methods

### Schistosoma japonicum samples

Adults of *S. japonicum* were available from a previous multilocus enzyme electrophoretic (MEE) study^21^ and had been stored at −70 °C until 1999, and then at −20 °C from 2000 to mid 2015. In brief, seven isolates of *S. japonicum* (designated Sj1-Sj7) originated from distinct endemic areas in China (Table 1). Cercariae were obtained from at least 10 infected snails (*Oncomelania hupensis*) collected from each province. *S. japonicum* adults were raised (45 days) in rabbits (n = 2 per province), each infected with 1,000 cercariae^21^. They were perfused from the mesenteric veins and washed extensively in physiological saline.

### Genomic DNA library construction, sequencing and pre-processing of reads

High molecular weight genomic DNA (10 μg) was isolated from pooled adult *S. japonicum* (i.e. male and female *en copula*; *n* = 10 pairs) representing each of the isolates Sj1 to Sj7 using a Chemagic DNA Tissue Extraction Kit (Chemagen). Total DNA amounts were determined using a Qubit fluorometer dsDNA HS Kit (Life Technologies), and DNA integrity was verified by agarose gel electrophoresis. High quality genomic DNA was used to construct short-insert (480 bp) genomic DNA libraries, which were then paired-end sequenced (2 × 100 base reads) utilising the TruSeq sequencing chemistry (Illumina) and the HiSeq 2500 sequencing platform (Illumina). High quality sequence data sets were produced by removing low quality bases (<25 Phred quality), adapters and reads of <50 nucleotides (nt) in length using the program Trimmomatic^66^.

### Assembly and curation of mt genomes

High quality paired-end reads were mapped to a reference mt genome sequence for *S. japonicum* (GenBank accession no. AF215860)^67^ using Bowtie2^68^. For each data set, paired-reads with at least one read aligned to the reference mt genome were extracted and *de novo*-assembled using the program SPADES v.3.10^69^ using a published mt genome sequence (accession no. AF215860)^67^ for *S. japonicum* as a “trusted” reference contig. Following assembly, paired-end reads were aligned to their corresponding mt genome using Bowtie2, and nucleotides and genome arrangement were verified using the program PILON^70^. Subsequently, each mt genome was annotated using an established pipeline^71^,^72^, and using the reference *S. japonicum* mt genome annotation (accession no. AF215860^67^) and echinoderm/flatworm mt code (NCBI genetic code - Table 9; http://www.ncbi.nlm.nih.gov/Taxonomy/Utils/wprintgc.cgi#SG9).

### Functional annotation of predicted protein sequences

*S. japonicum* proteins were compared by sequence homology (BLASTp; *E*-value ≤10^−5^) to proteins in databases representing *S. haematobium*^14^, *S. japonicum*^13^ and *S. mansoni*^30^; *Clonorchis sinensis*^46^ and *Opisthorchis viverrini*^47^; Swiss-Prot and TrEMBL within UniProtKB^73^ and Kyoto Encyclopedia of Genes and Genomes (KEGG)^74^. Conserved protein domains and gene ontology (GO) terms were identified for each inferred amino acid sequence using the program InterProScan^75^ employing the “–goterms” option. Each protein-encoding gene was assigned to a KEGG orthologous gene (KO) group using established methods^76^. Individual genes linked to one or more KO terms were assigned to known protein families and biological pathways using the KEGG BRITE and KEGG PATHWAY hierarchies using custom python scripts (available from the corresponding authors upon request). Putative signal peptide and transmembrane domains were predicted using the program Phobius^77^. In the final annotation, proteins inferred from genes were classified based on their homology (BLASTp; *E*-value <10^−5^) to sequences in (a) Swiss-Prot database, (b) the KEGG database, and (c) a recognised, conserved protein domain based on InterProScan analysis. Any predicted proteins without a match (*E*-value <10^−5^) in at least one of these databases were designated as hypothetical (or orphans).

### Identification and curation of nuclear genome polymorphisms

High quality reads were mapped to scaffolds (≥1000 bases) of the published nuclear (reference) genome of *S. japonicum* (SjRef; Bioproject PRJEA34885)^13^ using Bowtie2^68^. Duplicates were removed and insertion-deletion events were assessed and quality of read alignments were established using the sorted BAM files and PICARD tools (http://broadinstitute.github.io/picard) according to best practice (GATK guidelines)^78^. An MPILEUP format file was created from each BAM file using SAMtools^79^, and the frequencies of SNPs and insertion-deletion events (indels) were estimated using the program VarScan^80^. All SNPs and indels were contextualised within the current genome annotation (including exons, introns and intergenic elements) using snpEFF^81^ and a GFF annotation file available for the reference genome of SjRef^13^.

### Prediction of coding regions and identification of single copy orthologs

To predict the coding domains for each *S. japonicum* population, variant calls (SNPs only) inferred by VarScan^80^ were transferred to the genome reference using VCFtools (vcf-consensus)^82^, ignoring ambiguous positions (N) in the reference sequence of SjRef. Coding domains and amino acid sequences were extracted from each genome using GAG (http://genomeannotation.github.io/GAG) and the genome annotation for SjRef (Bioproject PRJEA34885)^13^. Single-copy orthologs (SCOs) between or among the genomes representing *S. japonicum* populations (Sj1-Sj7 and SjRef) and/or the outgroups *S. haematobium*^14^ and *S. mansoni*^30^ were defined using the program OrthoMCL^83^. Only SCOs of *S. japonicum* isolates (Sj1-Sj7) with an average, aligned read depth of >10 across all exonic regions and encoding the same start and stop codon positions as the reference genome (SjRef) were considered for further analysis. If the coding regions of *S. japonicum* genes homologous to biologically important genes in other schistosomes were only partial in the reference sequence (SjRef), the program PRICE^84^ and Illumina paired-end genomic reads sequenced in this study were used to extend the genomic region to obtain the full length gene for subsequent analyses.

### Phylogenetic analyses of nucleotide and amino acid data sets

To assess the genetic relationships among *S. japonicum* populations (Sj1-Sj7 and SjRef), mt genes or SCOs were aligned as individual nucleotide or inferred amino acid sequences using MAFFT^85^, selecting a minimum gap-free alignment length of 20 amino acids, and SCOs with at least one nucleotide or amino acid residue distinct from all others in the alignment. Nucleotide or amino acid sequence alignments were verified by eye, concatenated and then subjected to phylogenetic analyses using the methods Bayesian inference (BI) in MrBayes v.3.2.2^86^, maximum likelihood (ML) in the program RAxML v.8.0.24^87^ and maximum parsimony (MP) in PAUP* v.4.0 beta^88^, and including available reference (mt or nuclear) genomes for *S. japonicum*, and outgroups *S. haematobium* (nuclear), *S. mansoni* (nuclear) and *Schistosoma mekongi* (mt) (Table 2). For mt genome analysis, each protein-encoding gene region was partitioned and the HKY^89^ nucleotide substitution model was selected for individual genes using the Bayesian Information Criteria (BIC) test in jModeltest v. 2.1.6^90^. Amino acid substitution models were inferred for each gene using MrBayes by creating a consensus from available amino acid models (aamodelpr = mixed). For the analysis of nuclear genomic data, due to computation limitations the general time reversible model of evolution with gamma distribution^91^ was applied to concatenated SCO protein-encoding domains, and MrBayes created a consensus from available amino acid models (aamodelpr = mixed) for amino sequence alignments. For nucleotide and amino acid data sets, rates of reversible rate matrix, stationary state frequencies, shape of scaled gamma-distribution of site rates, partition-specific rate multiplier, topologies apropriori and branch lengths were unchanged from the default MrBayes v.3.2.2^86^ recommendations. Trees were constructed using sequence data for coding domains or proteins, employing the Monte Carlo Markov chain method (nchains = 4) over 100,000 (nuclear genome) or 2,000,000 (mt genome) generations, with every 100th (nuclear genome) or 200th (mt genome or primer set) tree being saved; 25% (mt) or 50% (nuclear) of the first saved trees were discarded to ensure a stabilisation of the nodal split frequencies. Consensus (50% majority rule) trees were constructed from all remaining trees, with nodal support expressed as a posterior probability (pp).

For ML, the same concatenated mt nucleotide and amino acid alignments were subjected to phylogenetic analysis using the general time reversible (GTR)^91^ and mtREV^92^ evolution models, respectively; concatenated alignment blocks were bootstrapped 200,000 times in RAxML to infer nodal support values. Concatenated SCO nucleotide and amino acid alignments were subjected to ML analysis using the general time reversible (GTR)^91^ and JTT^93^ evolution models, respectively and setting four discrete rate categories; concatenated alignment blocks were bootstrapped 100 times in RAxML^87^ to infer nodal support values. For mt and nuclear genome data sets, MP analyses were performed using heuristic searches, utilising tree bisection and reconnection (TBR), for each concatenated sequence alignment. By preserving branch lengths, the concatenated sequence blocks were bootstrapped 1,000 times using PAUP*^88^. The resultant, bootstrapped trees were then subjected to analysis in the program SumTrees in the DendroPy v.3.12.0^94^ python library to produce a consensus tree and to infer the nodal support values. The consensus trees were drawn and labeled using the program Figtree v.1.4 (http://tree.bio.ed.ac.uk/software/figtree/).

### Identification of the minimum, phylogenetically informative SCO set

Well-aligned sequence blocks were employed to identify corresponding exonic regions (600–1200 bp). Then, short (20–22 nt), conserved sequences were identified as PCR primers (5′ and 3′) in these regions using the program Primer3^95^. To establish which sets of exonic regions would produce a tree with the same topology as the final consensus tree for all SCOs among all populations, all possible combinations of two regions (designated pairs) were subjected to phylogenetic analysis using the ML-based software RAxML^87^. Trees matching the exact topology of the final consensus tree using all SCOs (cf. *Phylogenetic analyses of nucleotide and amino acid data sets*) were studied using Robinson-Foulds metric^96^ implemented in DendroPy^94^ python library, and examined further for robustness using 100-fold bootstrapping in the program RAxML^87^, employing a minimum nodal support value of 70%. Exonic regions fulfilling this stringent criteria were then exhaustively run in triplets and quartets resulting using 100-fold bootstrapping in RAxML. Finally, the best combination of four concatenated amplicons, with >92% nodal support, was selected as the minimum, informative sequence set required for phylogenetic reconstruction of the intraspecific *S. japonicum* tree. Phylogenetic analysis was the same as for mt data sets, except that a nucleotide substitution model was used.

### Selection of variable and invariable gene sets

Individual nucleotide and amino acid sequences representing all SCOs of individual *S. japonicum* populations were compared in a pairwise manner with their one-to-one orthologs in the *S. japonicum* reference genome of SjRef^13^. The nucleotide and amino acid identities as well as similarities were estimated for each pairwise comparison. SCOs were first ranked from least to most variable according to their mean nucleotide identity and standard deviation of mean nucleotide identity. Variable SCOs were defined as those SCO groups with one more SCO with ≤98% nucleotide identity to the SjRef-coding domain. Invariable SCOs were defined as those SCO groups with all pairwise alignments sharing ≥99.8%. nucleotide identity to the SjRef-coding domain. Enriched protein families and biological pathways were defined for selected genes sets using the Fisher’s exact test employing a custom script and linking data to KEGG biological pathway, KEGG BRITE hierarchy and gene ontology (GO) databases.

### *In silico* modeling of protein structure

Amino acid sequences of individual *S. japonicum* populations homologous to *S. mansoni* tetraspanin 2 (*Sm*-TSP2) were aligned using MAFFT^85^. The protein structure of *S. japonicum* TSP2 (*Sj*-TSP2) protein regions that aligned with the *Sm*-TSP2 extracellular 2 domain (EC2)^38^ were then modeled using I-TASSER^97^, and the resolved protein structure of *Sm*-TSP2-EC2 domain as a template (RCSB accession no. 2M7Z)^38^,^97^. Protein structure models were aligned and visualised using Chimera^98^,^99^,^100^,^101^,^102^.

## Additional Information

**How to cite this article**: Young, N. D. *et al*. Exploring molecular variation in *Schistosoma japonicum* in China. *Sci. Rep*. **5**, 17345; doi: 10.1038/srep17345 (2015).

## Supplementary Material

Supplementary Figures

Supplementary Table 1

Supplementary Table 2

Supplementary Table 3

Supplementary Table 4

Supplementary Table 5

Supplementary Table 6

Supplementary Table 7

Supplementary Table 7

Supplementary Table 8

## Figures and Tables

**Table 1:** Summary of concatenated mitochondrial and nuclear coding domain alignments and results of phylogenetic analyses.

	**Number of genes**	**Aligned character positions**	**Constant characters**	**Informative characters (%)**^b^	**Un-informative characters**	**Bayesian inference likelihood estimates:PSRF**^c^
Nucleotide – coding only						
Mitochondrial	12	10,341	7841	148 (1.43)	1024	−23,185:1
Nuclear^a^	4946	9,947,586	8,149,863	925,719 (9.31)	872,004	−21,562,691:1
PCR primer set (4 exons)	4^d^	3,378	3,314	43 (1.27)	21	−5109:1
Inferred protein translations						
Mitochondrial	12	3438	2375	39 (1.13)	1024	−15,788:1
Nuclear^a^	4946	3,315,862	2,613,069	335,882 (10.13)	366,911	−13,453,841:1.6
PCR primer set (4 exons)	4^d^	1,126	1,102	15 (1.33)	9	−3432:1

^a^OrthoMCL single copy orthologues among *S. japonicum*, *S. haematobium* and *S. mansoni*.

^b^Positions with polymorphic characters supported in two or more species.

^c^Average potential scale reduction factor (PSRF).

^d^Four genes; each represented by single protein-coding exon that can be PCR-amplified (see Supplementary Table 8).

**Table 2:** Single nucleotide polymorphisms (SNPs) recorded following the mapping of genomic sequence read data to the reference genome for *Schistosoma japonicum* (SjRef)^13^.

**Isolate code**	**Origin(County, Province)**	**Number of SNPs called**	**Intergenic SNPs**	**Exonic SNPs**	**Intronic SNPs**	**Non-synonymous SNPs**	**Synonymous SNPs**	**Sequencing depth within coding domains**^a^
Sj1	Jiashan, Zhejiang	7,156,718	4,924,071 (68.80%)	114,887 (1.61%)	1,888,984 (26.39%)	60,072	55,206	35.98 ± 42.42
Sj2	Guichi, Anhui	7,336,399	5,063,834 (69.02%)	114,136 (1.56%)	1,920,936 (26.18%)	59,157	55,378	42.94 ± 37.71
Sj3	Yongxia, Jiangxi	6,936,669	4,784,895 (68.98%)	107,446 (1.55%)	1,820,674 (26.25%)	55,316	52,507	39.80 ± 33.21
Sj4	Wuhan, Hubei	7,382,882	5,090,987 (68.96%)	116,471 (1.58%)	1,938,106 (26.25%)	60,981	55,886	43.73 ± 40.64
Sj5	Yueyang, Hunan	7,319,896	5,044,716 (68.92%)	115,692 (1.58%)	1,924,551 (26.29%)	60,477	55,612	40.19 ± 40.69
Sj6	Tianquan, Sichuan	6,879,937	4,767,718 (69.30%)	108,994 (1.58%)	1,784,457 (25.94%)	57,977	51,389	38.28 ± 52.02
Sj7	Dali, Yunnan	7,509,073	5,196,811 (69.21%)	121,775 (1.62%)	1,948,205 (25.94%)	64,473	57,725	39.35 ± 38.35

^a^Average ± standard deviation.

**Table 3:** Single nucleotide polymorphisms (SNPs) recorded in the coding domains of 4,413 single-copy orthologs (SCOs) among Schistosoma japonicum, S. haematobium and S. mansoni.

**Isolate code**	**Origin(County, Province)**	**Total intronic SNPs**	**Intronic SNPss per 1 kb**^a^	**Total exonic SNPs**	**Exon SNPs per 1 kb**^a^	**Non-synonymous SNPs**	**Synonymous SNPs**
Sj1	Jiashan, Zhejiang	747,394	14.5 ± 12.0	40,677	6.5 ± 6.1	18,999	21,678
Sj2	Guichi, Anhui	761,202	14.6 ± 12.5	40,033	6.4 ± 6.4	18,344	21,689
Sj3	Yongxia, Jiangxi	718,704	13.9 ± 12.2	37,438	6.0 ± 6.1	17,035	20,403
Sj4	Wuhan, Hubei	768,044	14.9 ± 12.4	41,210	6.6 ± 6.3	19,171	22,039
Sj5	Yueyang, Hunan	762,258	14.8 ± 12.4	40,521	6.5 ± 6.2	18,658	21,863
Sj6	Tianquan, Sichuan	697,639	13.5 ± 12.1	37,273	5.9 ± 6.1	17,550	19,723
Sj7	Dali, Yunnan	764,339	14.9 ± 13.0	42,333	6.8 ± 6.8	19,931	22,402

^a^Average ± standard deviation.
