# Supplementary material for: Exploring molecular variation in Schistosoma japonicum in China
Source: Sci Rep. 2015 Dec 1;5:17345. doi: 10.1038/srep17345 (PMC4664899; doi:10.1038/srep17345)
Supplement: Supplementary Figures [file srep17345-s1.pdf]

SUPPLEMENTARY FIGURES

Exploring molecular variation in *Schistosoma japonicum* in China

Neil D. Young[1], Kok-Gan Chan[2], Pasi K. Korhonen[1], Teik Min Chong[2], Robson Ee Han Jen[2], Namitha Mohandas[1], Anson V. Koehler[1], Yan-Lue Lim[2], Andreas Hofmann[1,3], Aaron R. Jex[1], Baozhen Qian[1], Neil B. Chilton[3], Geoffrey N. Gobert[4], Donald P. McManus[4], Patrick Tan[5,6], Bonnie L. Webster[7], David Rollinson[7], Robin B. Gasser[1]

[1] The University of Melbourne, Pathogen Genomics and Genetics Program, Parkville, Victoria 3010, Australia. [2] ISB (Genetics and Molecular Biology), Faculty of Science, The University of Malaya, Kuala Lumpur 50603, Malaysia,. [3] Structural Chemistry Program, Eskitis Institute for Drug Discovery, Griffith University, Brisbane, Queensland 4111, Australia. [4] Department of Biology, University of Saskatchewan, Saskatoon S7N 5E2, Canada. [5] QIMR Berghofer Medical Research Institute, Brisbane, Queensland 4006, Australia, [6] Genome Institute of Singapore, 60 Biopolis Street, Singapore 138672, Republic of Singapore. [7] Cancer and Stem Cell Biology, Duke-NUS Graduate Medical School, Singapore 138672, Republic of Singapore. [8] The Natural History Museum, London SW7 5BD, United Kingdom. Correspondence and requests for materials should be addressed to N.D.Y. (email: nyoung@unimelb.edu.au) or to R.B.G. (email: robinbg@unimelb.edu.au).

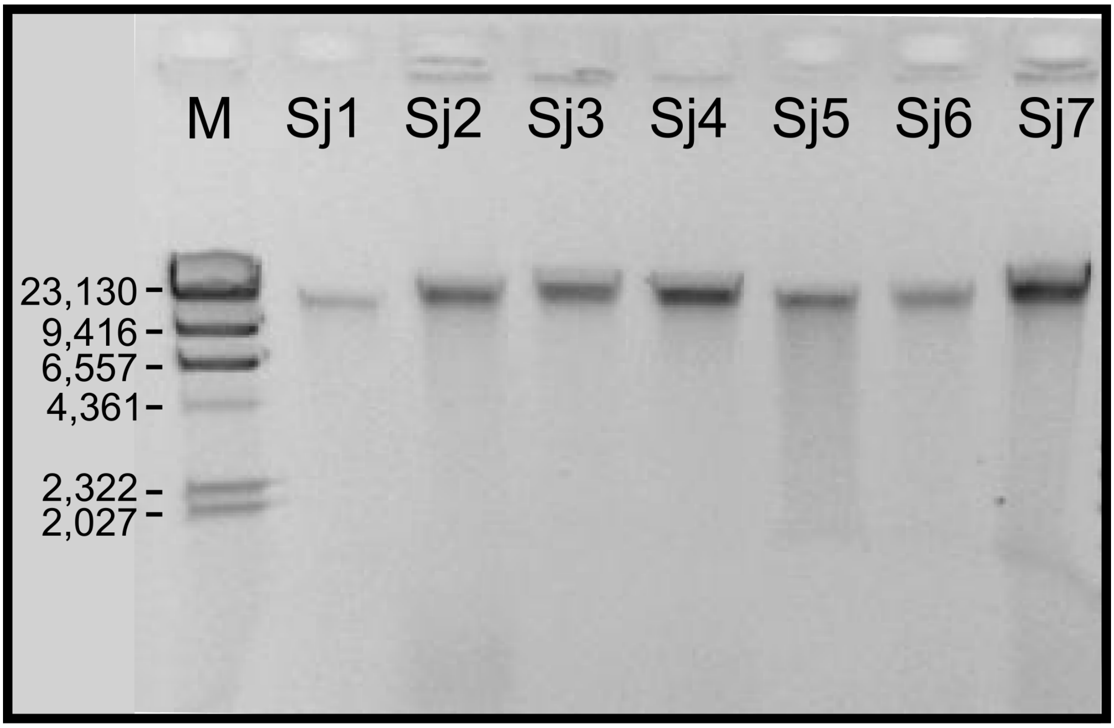

Supplementary Figure 1 | Integrity of archived *Schistosoma japonicum* genomic DNA. Genomic DNA was isolated from pooled adult *S. japonicum* (i.e. male and female *en copula*; *n* = 10 pairs) representing each of the populations Sj1 to Sj7. Genomic DNA samples were subjected to electrophoresis in an agarose gel (0.7%); Lambda DNA/*Hind*III was used as a molecular weight marker (M).

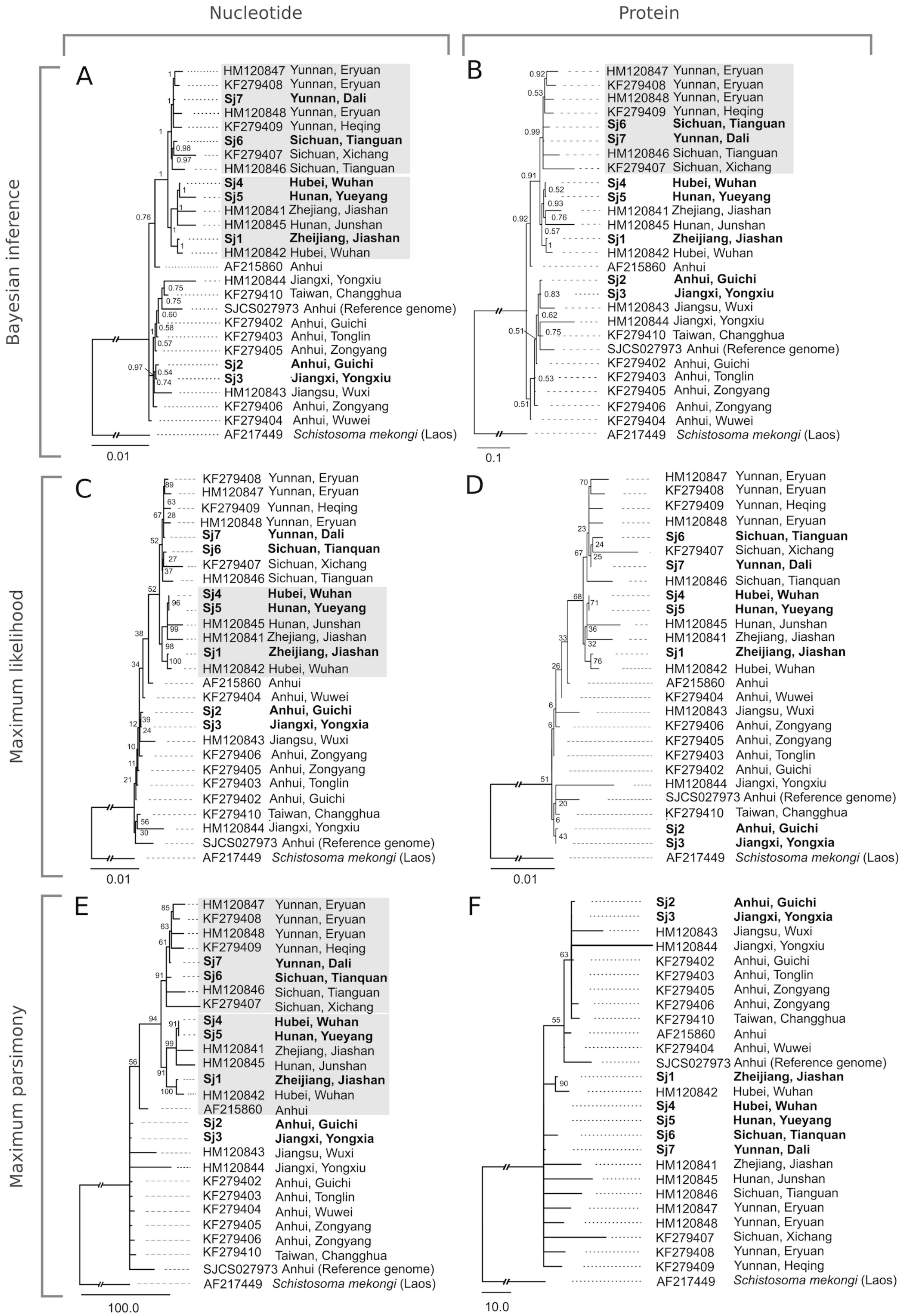

Supplementary Figure 2 | Phylogenetic relationships of *Schistosoma japonicum* populations based on analyses of data for 12 protein-encoding mitochondrial genes. The consensus trees from separate analyses of nucleotide (A, C and E) or amino acid (B, D and F) sequence data using the Bayesian inference (A and B), maximum likelihood (C and D) and maximum parsimony (E and F) tree-building methods. Posterior probabilities (pp) or nodal support values are indicated. Groups with strong nodal support are highlighted in grey. GenBank database accession numbers of individual gene sequences are at the ends of individual branches.

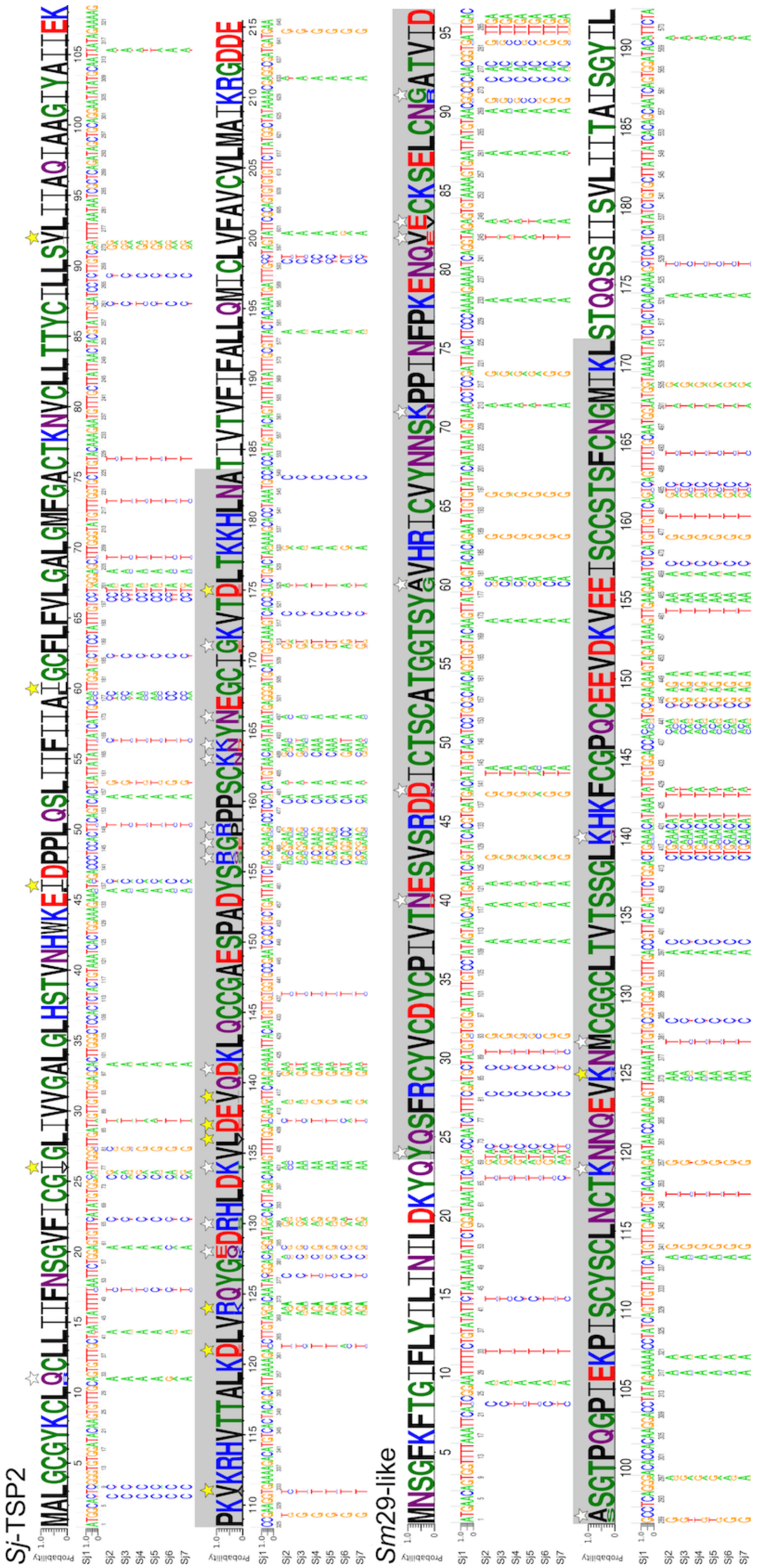

Supplementary Figure 3 | Genetic variability in immunogenic proteins among seven distinct populations of *Schistosoma japonicum*. Alignment of the nucleotide sequences encoding a consensus tetraspanin (*Sj*-TSP2) (A; Sjp_0118220) and *Sm*29-like (B; Sjp_0041250) protein among individual populations. Nucleotide logos represent the frequency of bases at sites containing single nucleotide polymorphisms (SNPs) for individual populations; Amino acid logos represent the consensus sequence for all seven populations. SNPs associated with a similar (yellow star) or distinct (white star) amino acid alteration are indicated. Each amino acid logo is coloured according to its chemical characteristics: polar residues (G, S, T, Y & C) are green, neutral (Q & N) are purple, basic (K, R & H) are blue, acidic (D & E) are red, and hydrophobic (A, V, L, I, P, W, F & M) are black. Immunogenic domains tested in vaccination experiments against *S. mansoni*[36] are highlighted in grey.
